# Dysglycemia but not lipids is associated with abnormal urinary albumin excretion in diabetic kidney disease: a report from the Kidney Early Evaluation Program (KEEP)

**DOI:** 10.1186/1471-2369-13-104

**Published:** 2012-09-07

**Authors:** Subhasish Bose, Andrew S Bomback, Nehal N Mehta, Shu-Cheng Chen, Suying Li, Adam Whaley-Connell, Joseph Benjamin, Peter A McCullough

**Affiliations:** 1 Department of Nephrology, Temple University Hospital, Philadelphia, PA, USA; 2 Division of Nephrology, Columbia University College of Physicians and Surgeons, New York, NY, USA; 3 Cardiovascular Institute, University of Pennsylvania School of Medicine, Philadelphia, PA, USA; 4 Chronic Disease Research Group, Minneapolis Medical Research Foundation, Minneapolis, MN, USA; 5 Dept of Internal Medicine, Division of Nephrology, Harry S Truman VA Medical Center and University of Missouri-Columbia School of Medicine, Columbia, MO, USA; 6 St. John Providence Health System, Providence Park Heart Institute, Novi, MI, USA

**Keywords:** Chronic Kidney Disease, Diabetes Mellitus, Proteinuria, Dyslipidemia, Glycosylated hemoglobin

## Abstract

**Background:**

The relationship between glycemic control and lipid abnormalities with urinary albumin-creatinine ratio (ACR) in chronic kidney disease (CKD) patients with diabetes mellitus (DM) is unknown. We sought to investigate the association of dyslipidemia and glycemic control with levels of albuminuria in the National Kidney Foundation (NKF) Kidney Early Evaluation Program (KEEP) participants with DM and CKD stage 3 or higher.

**Methods:**

We performed a cross-sectional study of 6639 eligible KEEP patients with DM and CKD Stage 3 to 5 from June 2008 to December 2009. Multivariate logistic regression was used to evaluate the association of lipid parameters (per 10 mg/dl change in serum level) and glycosylated hemoglobin (HbA1c) values with three degrees of albuminuria normo (<30 mg⁄g), micro (30 to 300 mg⁄g) and macro (>300 mg⁄g).

**Results:**

2141 KEEP participants were included. HbA1c levels were strongly associated with micro-albuminuria (compared to normo-albuminuria) and macro-albuminuria (compared to normo-albuminuria and micro-albuminuria). Each 1.0% increase in HbA1c increased the odds of micro-albuminuria by 32% (OR 1.32, 95% CI 1.23-1.42) and the odds of macro-albuminuria (vs. microalbuminuria) by 16% (OR 1.16, 95% CI 1.05-1.28). Only increases in serum HDL were associated with decreased odds of micro-albuminuria; otherwise, the association between other components of the serum lipid profile with urinary ACR did not reach statistical significance.

**Conclusion:**

In this cross-sectional study of 2141 KEEP participants with DM and CKD stages 3–5, overall glycemic control but not lipids were associated with abnormal urinary albumin excretion, a marker of increased risk for progressive disease.

## Background

Prevalence of chronic kidney disease (CKD) is increasing in the United States from approximately 12.7% reported in the National Health and Nutrition Examination Survey (NHANES) III (1988–1994) to 15.1% reported in the 2009 United States Renal Data System (USRDS) Annual Data Report, based on NHANES (2003–2006) data. CKD is a major cause of cardiovascular disease (CVD) morbidity and mortality worldwide and is now widely recognized as an independent risk for the development of CVD [1]. The relationship between CKD and CVD is complex but appears to be bidirectional, where therapy directed at improving natural history of one generally improves prognosis of the other. Specifically, CVD outcomes have been shown to improve with the treatment of risk factors commonly found in association with CKD progression including hypertension, diabetes mellitus (DM), and dyslipidemia [2].

Increasing albuminuria is a recognized predictor for CVD. Even mild elevations in the range of micro-albuminuria (30–299 mg/day) are associated with atherogenic lipoprotein abnormalities that promote endothelial dysfunction and CVD [3-5]. Serum lipid abnormalities have been established as a strong risk factor for CVD in the general population and are associated with advanced DM-related CKD and nephrotic range proteinuria. There is evidence that lipid abnormalities are associated with urinary albumin excretion (UAE) in patients with diabetes [6,7]. Increased UAE was found to be associated with ApoB-containing lipoproteins in patients with diabetes and the phenotype of hypertriglycerides/hyper-ApoB [6].

Our understanding of the lipid abnormalities associated with CKD and proteinuria are limited to correlative studies and targeting reductions in LDL. In this context, the relationship between dyslipidemia and diabetic kidney disease is derived from studies on HMG-coA reductase inhibition reducing proteinuria. However, limited data suggest that elevations in triglycerides have been implicated as a potential risk factor for progression of diabetic kidney disease [7]. Even less attention has been paid to the role of low versus high HDL parameters. We hypothesized that increasing levels of proteinuria would be associated with alterations in dyslipidemia and dysglycemia in patients with DM and CKD. We thus investigated the relationship between proteinuria, dyslipidemia, and dysglycemia in participants in the National Kidney Foundation (NKF)-Kidney Early Evaluation Program (KEEP).

## Methods

### NKF-KEEP

KEEP is a free community-based health-screening program that targets populations aged ≥18 years at high risk of kidney disease, defined as history of diabetes or hypertension or a first order relative with diabetes, hypertension, or kidney disease, as has been described previously. Screening methods have been reported previously [8]. Since August 2000, the program has screened ≥128,000 participants from 50 states and the District of Colombia. The KEEP database has been fully described in previous reports [9-12]. The Human Subjects Committees at the University of Minnesota approved this study. All procedures were in accordance with the Helsinki Declaration of 1975 as revised in 1983 and Title 45, U.S. Code of Federal Regulations, Part 46, Protection of Human Subjects, Revised November 13, 2001. Prior to enrollment, participants provided written informed consent. All authors were granted free access to the database.

This study included a total of 6639 eligible KEEP patients from June 2008 to December 2009 with HbA1c values. After excluding non-diabetic patients, non-CKD patients, and patients with missing values of urinary albumin-creatinine ratio (ACR), the total sample size was 2141.

### Definitions

Estimated glomerular filtration rate GFR (eGFR) was calculated using the 4-variable Modification of Diet in Renal Disease (MDRD) study equation, and serum creatinine was calibrated by the Cleveland Clinic Research Laboratory [13]. We would like to re-emphasize that CKD stage 3 was defined using KDOQI/KDIGO criteria as an NKF initiative with an estimated GFR <60 ml/min per 1.73m^2^***or*** >60 with an albumin to creatinine ratio >30mg /g. ACR were calculated from urine samples and recorded as <30 mg⁄g, 30 to 300 mg⁄g, or >300 mg⁄g. Microalbuminuria was defined as ACR of 30–300 mg/g, and macroalbuminuria, as ACR >300 mg/g. CKD was defined by an eGFR <60 mL⁄min⁄ 1.73 m^2^ or eGFR >60 with an ACR >30 mg/g. Other definitions included (1) hypertension: average systolic blood pressure >129 mm Hg or diastolic blood pressure >84 mm Hg (as per ATP III criteria for hypertension in association with dyslipidemia, glucose intolerance and abdominal obesity in metabolic syndrome) or a self-reported history of hypertension or patients taking blood pressure–lowering medication; and (2) dyslipidemia: triglyceride level >150 mg⁄dL or total cholesterol >200 mg⁄dL; (3) Diabetes mellitus: self-reported diabetes mellitus, retinopathy, and taking diabetic medications (including insulin). Other measures, including education level, tobacco and alcohol uses, and family history of diseases, were self-reported. Blood pressure, height, weight, and waist circumference were directly measured for all participants.

### Statistical Analysis

Demographic and baseline characteristics of the study population based on quartiles of glycosylated hemoglobin (HbA1c) were compared. Values were expressed as frequency (percent) unless noted otherwise. KEEP health screening results for the participants were also categorized by HbA1c values. We performed multivariate logistic regression analyses to evaluate individual association of lipid parameters (per 10 mg/dl change in serum level) and HbA1c values with ACR, adjusting for risk factors such as age, gender, race, status of hypertension and smoking. These associations were compared across different degrees of ACR: normo-albuminuria (<30 mg⁄g), micro-albuminuria (30 to 300 mg⁄g) and macro-albuminura (>300 mg⁄g).

## Results

The final eligible sample size included 2141 KEEP participants with diabetes and CKD stage 3–5 (Table 1). Participants were grouped into 4 quartiles of glycemic control delineated by HbA1c values: ≤6.2%, 6.3-6.9%, 7.0-8.1%, and ≥8.2%. The participants were predominantly female (65%) and non-hispanic white (57.5%) with a median age of 66 years (range 58–75). Participants with the worst glycemic control were significantly younger than participants with the best glycemic control. African-American and Hispanic participants were significantly over-represented in the highest quartile of HbA1c values. There was a high prevalence of concomitant hypertension (93%) in this cohort, yet only 144 participants (6.7%) self-reported current cigarette smoking. Mean serum creatinine and estimated GFR for the cohort were 1.2 mg/dl and 59.2 ml/min/1.73 m2, respectively. Roughly half (51%) of the participants had abnormal UAE, defined as ACR ≥ 30 mg/g. This prevalence rose significantly across quartiles of HbA1c, with participants in the highest quartile (A1c ≥8.2%) having nearly double the prevalence of abnormal ACR as participants in the lower quartile (A1c ≤ 6.2%). In Table 1, we have presented the statistical comparison between the groups of KEEP participants with the lowest (Q1) and highest (Q4) quartiles of HbA1c. We have also provided the statistical comparison of clinical characteristics across all HbA1c quartiles in Additional file 1: Table S1.

**Table 1:** Clinical Characteristics of KEEP Participants, 2008–2009, by Level of Glycemic Control as Evident by HbA1c

		**Quartiles of Glycosylated Hemoglobin (HbA1c)**				**Compare Q1 & Q4 (*p *value) §**
	**All, *n***	**Q1**	**Q2**	**Q3**	**Q4**	
		**[4.5 to 6.2]**	**[6.3 to 6.9]**	**[7.0 to 8.1]**	**[8.2 to 18.0]**	
***n *(row percentage)**	**2141 (100)**	**576 (26.9)**	**564 (26.3)**	**482 (22.5)**	**519 (24.2)**	
Age †	66 (58–75)	69 (61–77)	72 (63–78)	67 (60–75)	59 (50–68)	<.0001
Sex						0.2123
Men	749 (35)	188 (32.6)	183 (32.5)	190 (39.4)	188 (36.2)	
Women	1392 (65)	388 (67.4)	381 (67.5)	292 (60.6)	331 (63.8)	
Race						<.0001
NHW	1233 (57.5)	382 (66.3)	354 (62.8)	291 (60.4)	206 (39.7)	
NHAA	511 (24)	120 (20.8)	129 (22.9)	113 (23.4)	149 (28.7)	
Hispanic	215 (10)	43 (7.5)	49 (8.7)	38 (7.9)	85 (16.4)	
Other race	182 (8.5)	31 (5.4)	32 (5.7)	40 (8.3)	79 (15.2)	
≥ High school education	1740 (81.2)	477 (83.5)	467 (83.8)	390 (82.1)	406 (79.0)	0.0546
Current smoker	144 (6.7)	43 (7.6)	29 (5.3)	22 (4.6)	50 (10.0)	0.1773
Hypertensive	1991 (93)	535 (92.9)	525 (93.1)	445 (92.3)	486 (93.6)	0.6171
Family history of kidney disease	301 (14.4)	81 (14.4)	72 (13.1)	70 (15.0)	78 (15.2)	0.7562
Urinary ACR≥30 mg/g	1093 (51)	234 (40.6)	235 (41.7)	236 (49.0)	388 (74.8)	<.0001

*Note:* Values are *n* (column percent) unless otherwise indicated.

*Abbreviations*: *HbA1c*, Glycosylated Hemoglobin; *KEEP*, Kidney Early Evaluation Program; *NHW*, Non-Hispanic White; *NHAA*, Non-Hispanic African American; *ACR*, Albumin-creatinine ratio.

† Median (IQR).

§ chi^2^ for categorical variables and t-test for continuous variables.

Health screening results categorized by overall glycemic control (HbA1c quartiles) of individual participants varied significantly (Table 2). When compared across different quartiles of HbA1c values (Additional file 2: Table S2), a number of measures of metabolic derangements, including measures of anthropometry (body mass index - BMI and waist circumference), serum total cholesterol, low-density lipid (LDL), triglyceride (TG), and elevated urinary ACR values had incremental increases with rising HbA1c. Although serum Low-density lipid (LDL) were within normal range tended to increase with HbA1c except in quartile 1. High-density lipid (HDL) values, as expected, decreased across increasing quartiles of HbA1c.

**Table 2:** Health screening results categorized by HbA1c: KEEP database (2008–2009)

		**Quartiles of Glycosylated Hemoglobin (HbA1c)**				**Compare Q1 & Q4 (*p *value) §**
	**All, *n***	**Q1**	**Q2**	**Q3**	**Q4**	
		**[4.5 to 6.2]**	**[6.3 to 6.9]**	**[7.0 to 8.1]**	**[8.2 to 18.0]**	
***n *(row percentage)**	**2141 (100)**	**576 (26.9)**	**564 (26.3)**	**482 (22.5)**	**519 (24.2)**	
SBP (mm of Hg) ‡	138 (124–150)	136 (123–149)	137 (123–151)	138 (124–149)	139 (128–151)	0.0942
DBP (mm of Hg) ‡	78 (70–86)	78 (70–85)	75 (68–84)	78 (70–86)	80 (72–89)	0.0001
PP (mm of Hg) ‡	59 (48–70)	58 (48–70)	60 (48.5-71)	60 (49–70)	58 (49–69)	0.3544
BMI (kg/m2)‡	31.2 (27.4-36.6)	29.8 (26.3-34.9)	31.1 (27.3-36.3)	31.6 (27.7-36.8)	33.2 (28.7-38.3)	<.0001
WC (cm)‡	42 (38–46)	41 (37–45)	42 (38–46)	42 (39–47)	44 (39–48)	<.0001
Serum creatinine	1.13 (0.95-1.34)	1.15 (0.99-1.37)	1.16 (0.99-1.37)	1.17 (0.96-1.36)	1.03 (0.85-1.28)	<.0001
Estimated GFR	55.4 (46.1-68.9)	54.4 (44.7-59.8)	53.0 (44.6-59.5)	54.8 (45.9-65.3)	63.0 (50.4-84.4)	<.0001
Serum TC (mg/dl) ‡	177 (150–207)	178.5 (151.5-201.5)	170.0 (146.5-199.0)	171.0 (146.0-203.0)	188.0 (159.0-226.0)	<.0001
Serum LDL (mg/dl) ‡	89.5 (67–114)	90.5 (69–113)	84 (64–109)	86 (66–110)	98 (73–128)	0.0002
Serum HDL (mg/dl) ‡	47 (38–57)	50 (41–61.5)	48 (39–57)	45 (38–54)	44 (37–55)	<.0001
Serum TG (mg/dl) ‡	170 (117–250)	150 (108.5-214.5)	164.5 (113–241)	169 (114–253)	201 (142–301)	<.0001
Urinary ACR (mg/g)						<.0001
< 30	1048 (49.0)	342 (59.4)	329 (58.3)	246 (51.0)	131 (25.2)	
30 -300	966 (45.1)	210 (36.5)	214 (37.9)	205 (42.5)	337 (64.9)	
> 300	127 (5.9)	24 (4.2)	21 (3.7)	31 (6.4)	51 (9.8)	
Serum Calcium (mg/dl) ‡	9.7 (9.4-10)	9.7 (9.4-10.0)	9.7 (9.4-10.0)	9.6 (9.3-9.9)	9.6 (9.3-9.9)	0.4078
Serum Phosphorus (mg/dl)‡	3.6 (3.2-4)	3.6 (3.2-4.0)	3.6 (3.2-4.0)	3.6 (3.2-3.9)	3.65 (3.2-4.0)	0.4301
Hemoglobin (g/dl) ‡	13.3 (12.2-14.3)	13.2 (12.1-14.3)	13.1 (12.1-14.0)	13.3 (12.2-14.4)	13.7 (12.4-14.7)	<.0001

*Note:* Values are *n* (column percent) unless otherwise indicated.

*Abbreviations*: *HbA1c*, Glycosylated Hemoglobin; *KEEP*, Kidney Early Evaluation Program; *SBP*, Systolic Blood Pressure; *DBP*, Diastolic Blood Pressure; *PP*, Pulse Pressure; *BMI*, Body Mass Index; *WC*, Waist Circumference; *TC*, Total Cholesterol; *LDL*, Low Density Lipoprotein; *HDL*, High Density Lipoprotein; *TG*, Triglyceride; *ACR*, Albumin-creatinine ratio.

‡ Median (IQR).

§ chi^2^ for categorical variables and t-test for continuous variables.

In multivariate regression analyses (Table 3) controlling for age, gender, race, presence of hypertension and tobacco sue, only increased HDL was associated with a small, but significant, decreased odds of micro-albuminuria (vs. normo-albuminuria). Otherwise, across the range of micro- and macro-albuminuria assayed in the KEEP screenings, no lipid component was associated with ACR. Conversely, increased HbA1c was consistently and strongly correlated with level of albuminuria, whether the comparison was made between micro- and normo-albuminuria (OR 1.32, 95% CI 1.23-1.42), macro-albuminuria and non macro-albuminuria (OR 1.26, 95% CI 1.14-1.38), or macro-albuminuria vs. micro-albuminuria (OR 1.16, 95% CI 1.05-1.28).

**Table 3:** Association of albuminuria with components of serum lipid panel and HbA1c

**Adjusted for age, gender, race, hypertension, and current smoker**	**Odds ratio (95% Confidence Interval)**
(A) Micro-albuminuria Vs normo-albuminuria group:	
Total Cholesterol (mg/dl) (per 10)	1.03 (0.94-1.13)
Serum LDL (mg/dl) (per 10)	1.00 (0.91-1.10)
Serum HDL (mg/dl) (per 10)	0.88 (0.78-0.99)*
Serum Triglyceride (mg/dl) (per 10)	0.99 (0.97-1.01)
HbA1c	1.32 (1.23-1.42)***
(B) Macro-albuminuria Vs Non macro-albuminuria (Normo + Micro) group:	
Total Cholesterol (mg/dl) (per 10)	0.89(0.71-1.10)
Serum LDL (mg/dl) (per 10)	1.13 (0.90-1.42)
Serum HDL (mg/dl) (per 10)	1.20 (0.94-1.53)
Serum Triglyceride (mg/dl) (per 10)	1.01 (0.98-1.04)
HbA1c	1.26 (1.14-1.38)***
(C) Macro-albuminuria Vs micro-albuminuria group:	
Total Cholesterol (mg/dl) (per 10)	0.90(0.73-1.11)
Serum LDL (mg/dl) (per 10)	1.11 (0.89-1.37)
Serum HDL (mg/dl) (per 10)	1.25 (0.98-1.59)
Serum Triglyceride (mg/dl) (per 10)	1.01 (0.98-1.05)
HbA1c	1.16 (1.05-1.28)**

* p < 0.05, **p < 0.01, ***p < 0.001.

## Discussion

In this cross-sectional analysis of over 2000 KEEP participants with DM and CKD stages 3–5, we report that poor glycemic control is associated with micro-albuminuria and macro-albuminuria. These same associations were not apparent for any measured lipid as abnormalities were not present. Given that abnormal UAE is felt to be a marker of significantly high-risk kidney disease for any level of GFR, including a much greater risk for both rapid progression to end stage renal disease and premature cardiovascular morbidity and mortality, these data corroborate focusing on glycemic control in CKD patients with diabetes.

Several reports have identified the presence of CKD in DM patients with normoalbuminuria [14,15], but development of microalbuminuria has been considered to be one of the first detectable signs of the classic course of diabetic nephropathy that leads to CKD and, eventually, CVD and end-stage renal disease (ESRD). Consequently, there is a pressing need to further explore the role of risk factors for abnormal urinary albumin excretion, which conceivably “gets the ball rolling” in the pathogenesis of diabetic kidney disease. Glycemic control and dyslipidemia are two leading candidates for such risk factors.

Glycemic control, as evidenced by HbA1c, has previously been shown to be an independent risk factor for the development of microalbuminuria in type 1 and 2 diabetes mellitus in prospective study of normoalbuminuric patients [16]. However, our understanding of the relationship between proteinuria and glycemic control is largely derived from investigations targeting glycemic reductions and using proteinuria as a measure of kidney function in populations with preserved or only mildly impaired renal function. Several large randomized controlled trials, including the Diabetes Control and Complications Trial in type 1 diabetes [17], the UK Prospective Diabetes Study [18], and the Kumamoto Study [19], indicate that tighter glycemic control can decrease the risk of nephropathy. Recently, the ADVANCE trial [20] documented in subjects with DM that strict glycemic control (mean HbA1_c_: 6.5%), in comparison with the standard control (mean HbA1_c_: 7.3%), is associated with a significant reduction in renal events, including onset of or worsening of nephropathy [hazard ratio (HR) 0.79; *p* = 0.006], new-onset microalbuminuria (HR 0.91; *p* = 0.02) and, in particular, development of macroalbuminuria (HR 0.70; *p* < 0.001).

However, information about the effect of strict glycemic control on outcome in diabetic patients with established CKD is very limited [21], making it difficult to extrapolate that these positive effects of intensive glycemic control are indeed present in DM patients with moderate to advanced CKD. Therefore, our finding that poor overall glycemic control as evident from HbA1c values is significantly associated with proteinuria strengthens our understanding of the relationship between diverse metabolic risks and CKD progression. This relationship remained significant even after adjusting for co-variables (e.g. hypertension, smoking status) that are known to influence albuminuria. The strength of association of HbA1c with ACR was reduced when compared between micro-albuminuria and macro-albuminuria groups in our study. This finding could be reflection of the fact that HbA1c levels might underestimate mean blood glucose levels in patients with CKD and especially in diabetic subjects with severe nephropathy as evident from macro-albuminuria [22,23].

Dyslipidemia has been shown to be independently associated with micro [24] and macrovascular [25] diseases. A link between dyslipidemia and nephropathy has been demonstrated by prospective studies in the past [26,27]. However, understanding lipid fractions measured in these studies were limited due to the lack of information obtained regarding lipid-lowering therapies as well as fasting status and the lack of association with kidney disease is likely driven by the relatively normal measures. However, the importance of lipid abnormalities in kidney disease can not be understated. Other studies on patients with type 1 diabetes suggest a differential association between lipid variables and kidney disease depending upon the duration of diabetes [28] or the stage of renal impairment [29]. More recently, serum Apo(B) and Lp(a) increases have been reported at the stages of microalbuminuria and macroalbuminuria, respectively, in a cohort of type 2 diabetic patients but triglyceridemia was significant throughout the three stages of albuminuria [30]. In addition, decreased eGFR has been shown to be independently associated with greater odds of having a low HDL level [31]. Despite all these statistical associations between dyslipidemia and albuminuria, it is uncertain whether impaired lipid metabolism in CKD patients contributes to the progression of kidney disease [32] or, instead, progression of CKD and onset of significant albuminuria itself causes dyslipidemia [33].

In our study, neither microalbuminuria, nor macroalbuminuria was associated with lipids in screened KEEP participants. This finding partly may be explained by only 6% of the study population having overt proteinuria. Our understanding of dyslipidemia in CKD is in more advanced stages of CKD (i.e. stages 4–5) or with more significant proteinuria. Interestingly, we also found that HDL is negatively associated with ACR when compared between normo-albuminuria and micro-albuminuria. This association was not sustained when compared between micro-albuminuria and macro-albuminuria groups, perhaps underscoring other metabolic adverse effects that may contribute to low HDL in patients with advanced CKD or macroalbuminuria [31].

Our study does have several limitations that should be noted. The cross-sectional nature prohibits inference on causation. Secondly, we rely on single measurements of all laboratory parameters as screening program. While HbA1c values should not significantly change on repeated measurements (assuming glycemic control remains constant), conceivably both urinary ACR values and, more noticeably, lipid measurements can be affected by time of testing and pre-testing diet (e.g. fasting status). In addition, HbA1c values may underestimate mean blood glucose levels in CKD patients, particularly those with CKD-associated anemia, although this should be a non-directional bias towards the null in this cohort in which all participants had CKD. Finally, we do not have medication data on these participants including lipid-lowering therapies, which may influence the interpretation of the presented data. For example, if we knew that all patients in this cohort were on cholesterol-lowering medications such as statins, then we could theoretically explain the lack of association between rising lipid abnormalities and urinary ACR by a protective effect of statins.

## Conclusion

In conclusion, our findings demonstrate a strong correlation of overall glycemic control and abnormal urinary albumin excretion in patients with CKD and DM. Absence of similar association with dyslipidemia underscores the paramount importance of sustained blood glucose control in this population at high risk for cardiovascular disease and end stage renal disease. Until further prospective studies clearly delineate a causal link between lipid abnormalities and outcomes, it appears prudent for practitioners to continue to address lipid abnormalities in this population but to keep sustained emphasis on glycemic control, too.

## Competing interests

The Kidney Early Evaluation Program (KEEP)^TM^ is a program of the National Kidney Foundation, Inc., and supported by Amgen, Abbott, Siemens, Astellas, Fresenius, Genzyme, LifeScan, Nephroceuticals, and Pfizer. The authors report no personal conflicts of interest.

## Authors’ contributions

SB carried out literature review, initial research proposal, manuscript writing and subsequent revisions. ASB revised initial proposal and participated in the revision of manuscript and response to reviewers. NNM participated in the revision of manuscript. SCC and SL carried out initial and additional statistical analysis. AWC participated in the revision of manuscript and contributed to the response to reviewers. JB participated in the revision of manuscript. PAMc helped in revising initial proposal and reviewed manuscript revisions. All authors read and approved the final manuscript.

## Pre-publication history

The pre-publication history for this paper can be accessed here:

http://www.biomedcentral.com/1471-2369/13/104/prepub

## Supplementary Material

Additional file 1 **Table S1.** Clinical Characteristics of KEEP Participants, 2008-2009, by Level of Glycemic Control as Evident by HbA1c. Click here for file

Additional file 2 **Table S2.** Health screening results categorized by HbA1c: KEEP database. Click here for file
