# Supplementary material for: Dysglycemia but not lipids is associated with abnormal urinary albumin excretion in diabetic kidney disease: a report from the Kidney Early Evaluation Program (KEEP)
Source: BMC Nephrol. 2012 Sep 7;13:104. doi: 10.1186/1471-2369-13-104 (PMC3480932; doi:10.1186/1471-2369-13-104)
Supplement: Additional file 1 — Table S1. Clinical Characteristics of KEEP Participants, 2008-2009, by Level of Glycemic Control as Evident by HbA1c. [file 1471-2369-13-104-S1.doc]

**Supplementary Table 1.** Clinical Characteristics of KEEP Participants, 2008-2009, by Level of Glycemic Control as Evident by HbA1c

|  |  | Quartiles of Glycosylated Hemoglobin (HbA1c) | | | | Trend test (*p* value) § |
| --- | --- | --- | --- | --- | --- | --- |
|  | All, *n* | Q1  [4.5 to 6.2] | Q2  [6.3 to 6.9] | Q3  [7.0 to 8.1] | Q4  [8.2 to 18.0] |
| *n* (row percentage) | 2141 (100) | 576 (26.9) | 564 (26.3) | 482 (22.5) | 519 (24.2) |
| Age † | 66 (58-75) | 69 (61-77) | 72 (63-78) | 67 (60-75) | 59 (50-68) | <.0001 |
| Sex |  |  |  |  |  | 0.0548 |
| Men | 749 (35) | 188 (32.6) | 183 (32.5) | 190 (39.4) | 188 (36.2) |  |
| Women | 1392 (65) | 388 (67.4) | 381 (67.5) | 292 (60.6) | 331 (63.8) |  |
| Race |  |  |  |  |  |  |
| NHW | 1233 (57.5) | 382 (66.3) | 354 (62.8) | 291 (60.4) | 206 (39.7) | <.0001 |
| NHAA | 511 (24) | 120 (20.8) | 129 (22.9) | 113 (23.4) | 149 (28.7) | 0.0031 |
| Hispanic | 215 (10) | 43 (7.5) | 49 (8.7) | 38 (7.9) | 85 (16.4) | <.0001 |
| Other race | 182 (8.5) | 31 (5.4) | 32 (5.7) | 40 (8.3) | 79 (15.2) | <.0001 |
| ≥ High school education | 1740 (81.2) | 477 (83.5) | 467 (83.8) | 390 (82.1) | 406 (79.0) | 0.0387 |
| Current smoker | 144 (6.7) | 43 (7.6) | 29 (5.3) | 22 (4.6) | 50 (10.0) | 0.2246 |
| Hypertensive | 1991 (93) | 535 (92.9) | 525 (93.1) | 445 (92.3) | 486 (93.6) | 0.7559 |
| Family history of kidney disease | 301 (14.4) | 81 (14.4) | 72 (13.1) | 70 (15.0) | 78 (15.2) | 0.5191 |
| Urinary ACR≥30 mg/g | 1093 (51) | 234 (40.6) | 235 (41.7) | 236 (49.0) | 388 (74.8) | <.0001 |

*Note:* Values are *n* (column percent) unless otherwise indicated.

Abbreviations: HbA1c, Glycosylated Hemoglobin; KEEP, Kidney Early Evaluation Program; NHW, Non-Hispanic White; NHAA, Non-Hispanic African American; ACR, Albumin-creatinine ratio.

† Median (IQR)

§ Two-sided Cochran-Armitage Trend Test for categorical variables and Linear test for continuous variables
