# Supplementary material for: Dysglycemia but not lipids is associated with abnormal urinary albumin excretion in diabetic kidney disease: a report from the Kidney Early Evaluation Program (KEEP)
Source: BMC Nephrol. 2012 Sep 7;13:104. doi: 10.1186/1471-2369-13-104 (PMC3480932; doi:10.1186/1471-2369-13-104)
Supplement: Additional file 2 — Table S2. Health screening results categorized by HbA1c: KEEP database. [file 1471-2369-13-104-S2.doc]

**Supplementary Table 2.** Health screening results categorized by HbA1c: KEEP database (2008-2009)

|  |  | Quartiles of Glycosylated Hemoglobin (HbA1c) | | | | Trend test (*p* value) § |
| --- | --- | --- | --- | --- | --- | --- |
|  | All, *n* | Q1  [4.5 to 6.2] | Q2  [6.3 to 6.9] | Q3  [7.0 to 8.1] | Q4  [8.2 to 18.0] |
| *n* (row percentage) | 2141 (100) | 576 (26.9) | 564 (26.3) | 482 (22.5) | 519 (24.2) |
| SBP (mm of Hg) ‡ | 138 (124-150) | 136 (123-149) | 137 (123-151) | 138 (124-149) | 139 (128-151) | 0.1049 |
| DBP (mm of Hg) ‡ | 78 (70-86) | 78 (70-85) | 75 (68-84) | 78 (70-86) | 80 (72-89) | <.0001 |
| PP (mm of Hg) ‡ | 59 (48-70) | 58 (48-70) | 60 (48.5-71) | 60 (49-70) | 58 (49-69) | 0.1719 |
| BMI (kg/m2)‡ | 31.2 (27.4-36.6) | 29.8 (26.3-34.9) | 31.1 (27.3-36.3) | 31.6 (27.7-36.8) | 33.2 (28.7-38.3) | <.0001 |
| WC (cm)‡ | 42 (38-46) | 41 (37-45) | 42 (38-46) | 42 (39-47) | 44 (39-48) | <.0001 |
| Serum creatinine | 1.13 (0.95-1.34) | 1.15 (0.99-1.37) | 1.16 (0.99-1.37) | 1.17 (0.96-1.36) | 1.03 (0.85-1.28) | <.0001 |
| Estimated GFR | 55.4 (46.1-68.9) | 54.4 (44.7-59.8) | 53.0 (44.6-59.5) | 54.8 (45.9-65.3) | 63.0 (50.4-84.4) | <.0001 |
| Serum TC (mg/dl) ‡ | 177 (150-207) | 178.5 (151.5-201.5) | 170.0 (146.5-199.0) | 171.0 (146.0-203.0) | 188.0 (159.0-226.0) | <.0001 |
| Serum LDL (mg/dl) ‡ | 89.5 (67-114) | 90.5 (69-113) | 84 (64-109) | 86 (66-110) | 98 (73-128) | 0.0003 |
| Serum HDL (mg/dl) ‡ | 47 (38-57) | 50 (41-61.5) | 48 (39-57) | 45 (38-54) | 44 (37-55) | <.0001 |
| Serum TG (mg/dl) ‡ | 170 (117-250) | 150 (108.5-214.5) | 164.5 (113-241) | 169 (114-253) | 201 (142-301) | <.0001 |
| Urinary ACR (mg/g) |  |  |  |  |  | <.0001 |
| < 30 | 1048 (49.0) | 342 (59.4) | 329 (58.3) | 246 (51.0) | 131 (25.2) |  |
| 30 -300 | 966 (45.1) | 210 (36.5) | 214 (37.9) | 205 (42.5) | 337 (64.9) |  |
| > 300 | 127 (5.9) | 24 (4.2) | 21 (3.7) | 31 (6.4) | 51 (9.8) |  |
| Serum Calcium (mg/dl) ‡ | 9.7 (9.4-10) | 9.7 (9.4-10.0) | 9.7 (9.4-10.0) | 9.6 (9.3-9.9) | 9.6 (9.3-9.9) | 0.1311 |
| Serum Phosphorus (mg/dl)‡ | 3.6 (3.2-4) | 3.6 (3.2-4.0) | 3.6 (3.2-4.0) | 3.6 (3.2-3.9) | 3.65 (3.2-4.0) | 0.7384 |
| Hemoglobin (g/dl) ‡ | 13.3 (12.2-14.3) | 13.2 (12.1-14.3) | 13.1 (12.1-14.0) | 13.3 (12.2-14.4) | 13.7 (12.4-14.7) | <.0001 |

*Note:* Values are *n* (column percent) unless otherwise indicated.

Abbreviations: HbA1c, Glycosylated Hemoglobin; KEEP, Kidney Early Evaluation Program; SBP, Systolic Blood Pressure; DBP, Diastolic Blood Pressure; PP, Pulse Pressure; BMI, Body Mass Index; WC, Waist Circumference; TC, Total Cholesterol; LDL, Low Density Lipoprotein; HDL, High Density Lipoprotein; TG, Triglyceride; ACR, Albumin-creatinine ratio.

‡ Median (IQR)

§ Two-sided Cochran-Armitage Trend Test for categorical variables and Linear test for continuous variables
